# Quantum Dots for Cancer Research: Current Status, Remaining Issues, and Future Perspectives

**DOI:** 10.7497/j.issn.2095-3941.2012.03.001

**Published:** 2012-09

**Authors:** Min Fang, Chun-wei Peng, Dai-Wen Pang, Yan Li

**Affiliations:** 1 Department of Oncology, Zhongnan Hospital of Wuhan University, Hubei Key Laboratory of Tumor Biological Behaviors & Hubei Cancer Clinical Study Center, Wuhan 430071, China; 2 Key Laboratory of Analytical Chemistry for Biology and Medicine (Ministry of Education), College of Chemistry and Molecular Sciences, and State Key Laboratory of Virology, Wuhan University, Wuhan 430072, China

**Keywords:** quantum dots, molecular imaging, multimodality probes

## Abstract

Cancer is a major threat to public health in the 21st century because it is one of the leading causes of death worldwide. The mechanisms of carcinogenesis, cancer invasion, and metastasis remain unclear. Thus, the development of a novel approach for cancer detection is urgent, and real-time monitoring is crucial in revealing its underlying biological mechanisms. With the optical and chemical advantages of quantum dots (QDs), QD-based nanotechnology is helpful in constructing a biomedical imaging platform for cancer behavior study. This review mainly focuses on the application of QD-based nanotechnology in cancer cell imaging and tumor microenvironment studies both *in vivo* and *in vitro*, as well as the remaining issues and future perspectives.

## Introduction

Cancer remains a global public health problem as the leading cause of death in developed countries and the second leading cause of death in developing countries^[^^1^^]^. It is a diverse group of diseases affecting a variety of tissues, but is generally characterized by the uncontrolled proliferation of abnormal cells, with the ability to invade surrounding tissues, and possibly metastasize. Over the past few decades, numerous studies have focused on the regulation of cell adhesion and cytoskeletal dynamics as the mechanisms of cancer invasion and progression^[^^2^^-^^4^^]^. However, they have largely failed to define the rate-limiting mechanisms that govern cancer invasion and progression, such as the dominant signaling pathway, receptor–ligand interactions, or protease–substrate interactions. At present, cancer invasion is regarded as a heterogeneous and adaptive process with a tumor microenvironment ^[^^5^^]^. The tumor microenvironment, composed of tumor stromal components, host cells, and adjacent supporting tissues, is an intrinsic element because of its dynamic interactions with the tumor for continued tumor growth and progression^[^^6^^,^^7^^]^. Once the tumor microenvironment responds to the tumor cells, components such as fibroblasts, endothelial cells, and macrophages could be activated and could release functional factors that promote or inhibit cancer invasion^[^^8^^-^^14^^]^. Thus, tumor cells influence the microenvironment and vice versa, jointly driving cancer progression in a reciprocal manner^[^^15^^,^^16^^]^. Thus, to intensively explain the mechanism of cancer invasion and progression, understanding the biological behavior during tumor progression is necessary, and the appropriate approach for understanding the biological behavior of cancer should be established.

Nanotechnology is a promising platform in cancer molecular imaging. Quantum dots (QDs) are being intensively studied as a novel probe for biomedical imaging both *in vitro* and *in vivo* because of their unique optical and electronic characteristics. To overcome the obstacles of QDs for biomedical imaging, the physicochemical properties of QDs such as size, shape, composition, and surface features have been extensively investigated^[^^17^^-^^23^^]^. When conjugated with bimolecular agents such as antibodies, peptides or other small molecules, QD-based probes can be used to target cancer molecules with high specificity and sensitivity. Thus, QD-based multiplexed molecular imaging can reveal the tempo-spatial relationship among molecules by simultaneously staining several tumor biomarkers. Several studies have shown that this method is essential for deciphering the molecular mechanism of cancer invasion and is useful for the study of tumor microenvironment^[^^20^^-^^22^^]^. In addition to the promising application for molecular imaging *in vitro*, QD-based multifunctional probes lead to the development of anti-cancer drug and siRNA delivery^[^^24^^]^, magnetic resonance imaging (MRI)^[^^25^^,^^26^^]^, multiplexed molecular cancer diagnosis, and *in vivo* imaging^[^^27^^-^^30^^]^. In this view, QDs can be used to monitor the dynamic changes of a tumor microenvironment, which would greatly contribute in the research of cancer invasion mechanism and guide better clinical personalized therapy. Therefore, compared with conventional imaging approaches, a molecular QD-based targeted nanoplatform offers various advantages. First, hundreds, thousands or even more imaging labels or combinations of labels for different imaging modalities can be attached to a single nanoparticle, which can lead to dramatic signal amplification. Second, multiple, potentially different, targeting ligands on the nanoparticle can provide enhanced binding affinity and specificity. Third, the ability to integrate a specific biomarker to bypass biological barriers can enable enhanced targeting efficacy. Ultimately, the combination of different targeting ligands, imaging labels, therapeutic drugs, and many other agents may allow the effective and controlled delivery of therapeutic agents in patients, which can be noninvasively monitored in real time.

In this review, we summarize the major advances in the application of QD-based nanotechnology for cancer research, including the detection of primary tumor *in vitro*, tumor imaging *in vivo*, study of tumor microenvironment for invasion, and progression and multimodality biomedical molecular targeting imaging, as well as the major remaining issues and future perspectives.

## Characteristics of QDs for Biomedical Application

QDs are semiconductor nanocrystals that range from 2 nm to 10 nm in diameter and consist of elements from groups II to VI or III to V. Given their special size and surface effect, QDs are one of the most promising nanocrystals with unique optical and chemical properties. QDs offer great advantages over traditional organic fluorescent dyes and present a number of beneficial characteristics for spectroscopy, such as high fluorescence intensity, long lifetime, and good resistance to photobleaching. The brightness of QD-based multifunctional probes affords high sensitivity for simultaneous cancer molecular imaging and targeted therapy. For spectrum application, the sensitivity of QD-based molecular imaging can be two to three orders larger than that of routine fluorescent dyes^[^^31^^]^ (**Table 1**). Furthermore, the fluorescence in near infrared (NIR) of NIR-QDs can be detected in deep tissues, making them suitable for *in vivo* imaging with high signaltobackground ratio^[^^17^^,^^18^^,^^23^^]^.

**Table 1:** Comparison of the characteristics and applications between traditional organic fluorophores and QDs.

Property	Traditional organic fluorophores^[^^32^^–^^35^^]^	Quantum dots^[^^29^^, ^^36^^–^^42^^]^
		
Chemical properties	Chemical resistance is often poor	Resistant to chemical degradation; sensitivity to pH determined by coatings
Size scale	Molecular, <0.5 nm	Colloidal, 1.5 nm to 10 nm diameter
Hydrodynamic radius	Small, <0.6 nm^a^	Variable, 1.4 nm to 40 nm^b^
Absorption spectra	Discrete bands, FWHM^c^, 35 nm^d^ to 80 nm to 100 nm^e^	Strong and broad
Emission spectra	Broad, red-tailed, and asymmetric, FWHM, 35 nm to 70 nm to100 nm	Narrow, symmetric, FWHM, 30 nm to 90 nm
Two-photon cross-section	10 GM to 500 GM	(2,000 to 47,700) GM^f^
Molar absorption coefficient	(10^3^ to 10^5^) cm^-1^mol^-1^L	(10^5^ to 10^6^) cm^-1^mol^-1^L
Quantum yield	Variable, 0.05 to 1.0	High, >20%^g^
Fluorescence lifetime	Short, <5 ns, mono-exponential decay	Long, >10 ns, typically multi-exponential decay
Solubility or dispersibility	Control by substitution pattern	Control via surface chemistry (ligands)
Thermal stability	Dependent on dye class; can be critical for NIR-wavelength dyes	High; depends on shell or ligands
Photostability	Usually poor	Excellent resistance to photobleaching; observation time of minutes to hours
Bioconjugation labels	Monovalent to multivalent labeling possible	Scaffolds; Monovalent conjugation can be challenging; distribution of multivalences often encountered
Applicability to single molecule analysis	Moderate; limited by photobleaching	Good; limited by blinking
Spectral Multiplexing	Possible	Ideal for multi-color experiments; up to five colors demonstrated
Multifunctionality	Difficult and few	Great potential
Toxicity	Variable, based on dye	Related to the heavy metal

^a^:Except for fluorescent proteins, GFP 4.6×2.4 nm cylindrical shape ^b^:Coating, ligand, and bioconjugate-dependent ^c^:FWHM, full width at half height of the maximum. ^d^:Dyes with resonant emission, such as fluoresceins, rhodamines and cyanines. ^e^:CT dyes. ^f^:Wavelength-dependent; GM: Goeppert–Mayer units ^g^:Ligand, coating and solvent-dependent

## Application of QD-based Nanotechnology for Cancer Research

### Detection of primary tumor *in vitro*

Since biocompatible QDs were introduced for imaging of cancer cells *in vitro* in 1998^[^^43^^,^^44^^]^, researchers have synthesized QD-based probes conjugated with cancer specific ligands, antibodies, or peptides for cancer imaging and diagnosis *in vitro*^[^^29^^,^^45^^-^^52^^]^. Compared with traditional immunohistochemistry (IHC), QD-IHC is more accurate and precise at low protein expression levels^[^^53^^-^^56^^]^ and can achieve quantitative detection which will provide much more information for personalized treatment^[^^53^^]^. With excellent performance on biomedical imaging, QD-based imaging has become one of the most promising technologies for early diagnosis of cancer^[^^57^^,^^58^^]^.

### Prostate cancer

A classic example of cancer detection was demonstrated by Gao et al.^[^^45^^]^ who labeled human prostate cancer cells based on the conjugate of QDs with an antibody for prostate specific membrane antigen (PSMA). Ruan et al.^[^^59^^]^ showed that QD-based immunolabelling has more stable photo-intensity compared with conventional fluorescent immunolabelling. Highly sensitive QD-based probes have been reported for multicolor fluorescence imaging of cancer cells *in vivo*^[^^42^^]^. Shi et al.^[^^60^^]^ showed the superior quality of QD-IHC compared with conventional IHC and also successfully realized simultaneous detection of androgen receptor and PSA in prostate cancer cells based on multiplexing QDs. The detection sensitivity of QD-based prostate cancer biomarkers can be enhanced by surface plasmon-coupled emission which has been introduced as a novel biosensing technology for detecting biosensors and biochips^[^^61^^,^^62^^]^. It can be a highly sensitive and efficient detection system for genomic and proteomic applications by rejecting background emission^[^^63^^]^.

### Breast cancer (BC)

**Figure 1:**
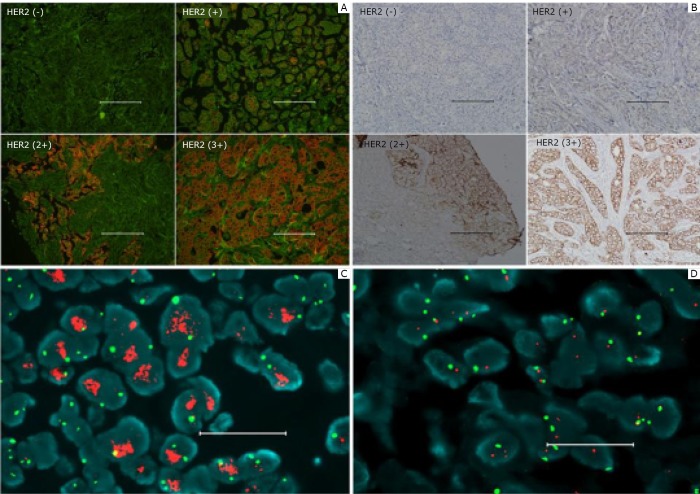
Accurate HER2 testing by QD–IHC. A: Specimens with different HER2 IHC scores detected by QD–IHC. B: Control for (A) by conventional IHC. C: FISH positive. D: Negative. Scale bar: 100 µm for (A) and (B); 20 µm for (C) and (D). Reproduced with permission from [55].

Human epidermal growth factor receptor 2 (HER2) is overexpressed in approximately 25% to 30% BC patients and has an important function in cancer progression. Recent studies have validated the value of HER2 detection for BC treatment and prognosis ^[^^64^^, ^^65^^]^. Compared with the golden standard method of fluorescence in situ hybridization (FISH), the advantages of QD-based IHC have been well documented since Wu et al.^[^^29^^]^ labeled HER2 on human BC cells (SK-BR-3) and mouse mammary tumor sections by QD-IgG conjugates for the first time, which was much easier, cheaper, less time-consuming, and could relieve the medical burden, especially for developing countries. Various studies have reported the successful detection for BC by QD-HER2 conjugates^[^^66^^]^. Yezhelyev et al.^[^^67^^]^ extended this approach to selectively label MCF-7 and BT-474 BC cells for HER2, epidermal growth factor receptor (EGFR), estrogen receptor (ER), progesterone receptor (PR), and mammalian target of rapamycin (m-TOR) by visible and NIR QDs which indicated that QD-based nanotechnology is an efficient approach to offer multiplexed cancer biomarker imaging in situ on intact tumor tissue specimens for tumor pathology study at the histological and molecular levels simultaneously^[^^20^^-^^22^^]^. Our group also conducted a series of studies of BC based on QD-IHC in HER2 detection and QD-based quantitative spectral analysis of HER2, ER, and PR^[^^54^^-^^56^^,^^68^^]^. Chen et al.^[^^55^^]^ successfully detected BC with QD-based probes which demonstrated that lower expression of HER2 could be clearly detected by QD-IHC compared with conventional IHC (**Figure 1**) and could also realize multiplexed QD-based detection simultaneously^[^^69^^]^. The results showed that BC can be divided into 5 subtypes with different 5-year survival rate. Thus, QD-based multiplexed imaging will provide more information for the individual events of tumor, personalized diagnosis, prognosis, and treatment.

### Ovarian cancer

QDs can also be used to detect the ovarian carcinoma marker CA125 in different types of specimens, such as fixed cells, tissue sections, and xenograft piece. Additionally, the photostability of QD signals is more specific and brighter than that of conventional organic dye^[^^70^^]^. Liu et al.^[^^71^^]^ synthesized pH-sensitive photoluminescent CdSe/ZnSe/ZnS QDs in SKOV-3 human ovarian cancer cells that are pH-dependent, suggesting applications for intracellular pH sensors. Kawashima et al. successfully targeted EGFR single-molecules in human ovarian epidermal carcinoma cells (A431)^[^^72^^]^.

### Gastrointestinal cancer

Bostick et al.^[^^73^^]^ detected the five biomarkers on the same tissue slide by QD-based multiplexed imaging, from which more biomarkers could be measured using multiple slides each stained with the five different biomarkers. They further proposed to construct a workflow for the quantitative analysis of each biomarker. The system was both efficient and convenient, such that it only took 7 h to analyze six biomarkers, which was advantageous for clinical application.

### Pancreatic cancer

QD-based imaging probes can target pancreatic cancer at a very early stage^[^^74^^,^^75^^]^ with the help of proteins/peptides directed against overexpressed surface receptors on cancer cells/tissues, such as the transferring receptor, antigen claudin-4, and urokinase plasminogen activator receptor^[^^76^^-^^78^^]^. CdSe/CdS/ZnS QDs with improved photoluminescence efficiency and stability as optical agents have been used for the imaging of pancreatic cancer cells using transferring and anti-Claudin-4^[^^79^^]^. Yang et al.^[^^75^^]^ used non-cadmium-based QDs as highly efficient and non-toxic optical probes for imaging live pancreatic cancer cells. Further bioconjugation with pancreatic cancer-specific monoclonal antibodies, such as anti claudin-4, to the functionalized InP/ZnS QDs, allowed specific *in vitro* targeting of pancreatic cancer cell lines. Lee et al.^[^^80^^]^ reported quantitative molecular profiling of biomarkers for pancreatic cancer with functionalized QDs. They obtained absolute quantitative values for the biomarker density in terms of the number of molecules per square micron on the cell surface, which is important because cancer cell populations are inherently heterogeneous. They also demonstrated highly selective targeting of molecular markers for pancreatic cancer with extremely low levels of nonspecific binding.

### *In vivo* tumor imaging

*In vivo* tumor imaging can directly demonstrate the evolution mechanism of tumor progression. More convincing evidence could be obtained from *in vivo* tumor imaging compared with *in vitro* molecular imaging. However, sensitive and specific imaging agents are urgently needed for high-quality *in vivo* tumor imaging and less biological impacts on the animal model. QD-based imaging agents can meet this demand by “enhanced permeability and retention” (EPR) or targeted molecular imaging. The principle of EPR-based tumor imaging is the leakiness of tumor blood vessels. Compared with normal tissues, tumor vasculature is quantitatively important, but irregular, leaky, dilated, and vascular endothelial cells are poorly aligned with large fenestrations^[^^81^^,^^82^^]^. The morphology results in increased leakage of macromolecules and nanocarriers out of the circulatory system into the tumor tissue by the EPR effect^[^^83^^,^^84^^]^. They finally accumulate in the tumor microenvironment because of the lack of effective lymphatic drainage. This EPR effect has inspired the development of a variety of nanotherapeutics and nanoparticulates for the imaging and treatment of cancer^[^^83^^, ^^85^^-^^87^^]^. Many studies have reported that non-targeted QDs can be used for cell trafficking^[^^88^^]^, vasculature imaging^[^^89^^]^, sentinel lymph node (SLN) mapping^[^^30^^,^^90^^,^^91^^]^, and neural imaging^[^^92^^]^.

SLN diagnosis contributes to operation strategy in cancer surgery. During lymph node metastasis, cancer cells first reach the SLN via lymph flow. The cancer cells should be detected with high sensitivity in the SLN connected to the tumor site to perform SLN biopsy effectively. For instance, the superiority of NIR QDs (emitting at 850 nm) has been demonstrated in SLN mapping, a common procedure in BC surgery, whereby the lymph node closest to the targeted organ is monitored for the presence of locally disseminated cancer cells. Recent studies have also reported on highly sensitive, real-time intra-operative SLN mapping of the gastrointestinal tract by NIR light and invisible fluorescent QDs^[^^76^^]^ with high background-to-signal ratio. In these studies, NIR QDs allowed image guidance throughout the entire procedure, virtually free of any background. The SLNs and their eventual removal were imaged in real time, without the need of traditional dyes or radioactive tracers^[^^30^^,^^93^^]^. Hikage et al.^[^^94^^]^ effectively detected metastatic gastrointestinal cancer cells in SLN with high sensitivity. So et al.^[^^95^^]^ used luciferase-conjugated QDs to obtain self-illuminating QDs, which totally eliminates the issue of tissue autofluorescence. Voura et al.^[^^49^^]^ also demonstrated that QDs can track different QD-tagged populations of cancer cells in the same animal by multiphoton laser scanning microscopy. This finding may contribute to our understanding of metastasis, which remains a fundamental barrier to the development of effective cancer therapy. Given the high sensitivity and penetration (approximately 1 cm below the skin surface) of NIR QD fluorescence, the application of QD-based SLN mapping allows the surgeon to define the tumor border accurately and minimize the size of the dissection^[^^30^^,^^38^^,^^51^^,^^94^^,^^96^^]^. This successful technology in preclinical studies represents a significant breakthrough, and further studies are advantageous to pave the way to clinical application.

QDs need to be effectively, specifically, and reliably directed to a specific organ or disease site without alteration to make them more beneficial for biomedical applications. Specific targeting can be achieved by attaching targeting molecules to the QD surface. After the first successful application of QDs *in vivo* by Akerman et al.^[^^97^^]^, studies based on QDs in mouse models for cancer imaging were subsequently conducted^[^^45^^,^^50^^,^^98^^,^^99^^]^. Gao et al.^[^^45^^]^ performed whole animal cancer imaging by QD-PSMA. The QD-PSMA was efficiently and uniformly distributed in the prostate tumor, indicating the potential for accurate prostate cancer diagnosis and real-time monitoring. Cai et al.^[^^50^^]^ targeted glioblastoma with NIR QD-peptide conjugate and showed high signal-to-background ratio and long duration of signals. Arginine-glycine-aspartic acid peptide-conjugated QDs have been used to target integrin αvβ3 in a murine xenograft model specifically^[^^50^^,^^100^^]^ because integrin αvβ3 is significantly upregulated in tumors, but not in normal tissues.

**Figure 2:**
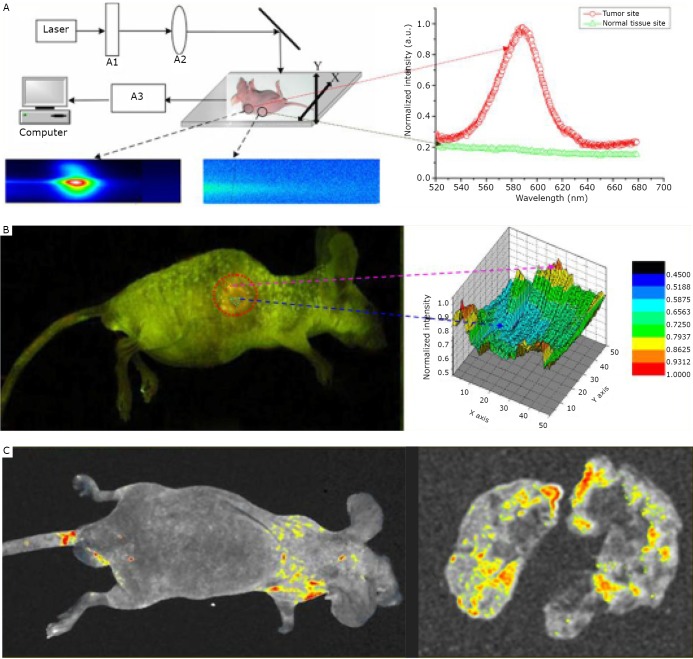
*In vivo* targeting and imaging of a lung metastasis model with QD-based nanotechnology. A: The imaging system for living animal models. B: *In vivo* targeted imaging of the subcutaneous tumor model and site-by-site spectra analysis of the tumor, which showed that the QD-labeled anti-a-fetoprotein monoclonal antibody probes per field were lower at the center than in the periphery of the tumor, indicating that tumor growth was not homogeneous and the peripheral site was more active. C: *In vivo* targeted imaging of liver cancer lung metastasis models. Reproduced with permission from [53].

In addition, our group developed a standard protocol for in vivo imaging of liver cancer xenograft animal models^[^^98^^,^^101^^-^^103^^]^. We successfully achieved animal imaging by injecting human hepatocellular carcinoma cell lines (HCCLM6) that overexpress alpha-fetoprotein (AFP) with antiAFP monoclonal antibody and QD-IgG probes. HCCLM6 has increased potential for lung metastasis, so it helps in the construction of a platform for the early monitoring of liver cancer metastasis (**Figure 2**)^[^^53^^]^.

However, not enough experimental evidence is available to support the conclusion that the tumor contrast observed was from active, rather than passive, targeting. Tracking the movement of a single QD-antibody conjugate (the total number of QD particles injected was approximately 1.2×10^14^) inside the tumor through a dorsal skinfold chamber was accomplished using a high-speed confocal microscope with a high-sensitivity camera^[^^46^^]^. This technique captured the specific delivery of a single QD particle that entered into circulation, extravasated into the interstitial space from the vasculature, bound to the tumor cell surface receptor, and reached the perinuclear region after it traveled on the intracellular rail protein.

Minimizing non-specific uptake of QDs by the reticuloendothelial system (RES) is necessary to maximize active targeting. Hence, proper surface modification of QDs must be designed to adjust the complicated anatomical structure and physiology in tissues and organ systems that create barriers for QDs^[^^101^^,^^104^^]^. For example, coating QDs with high-molecular weight poly-ethylene-glycol (PEG) molecules can reduce QD accumulation in the liver and bone marrow^[^^105^^]^. When using neutral methoxy-terminated PEG (mPEG) coating, results vary depending on the length of the PEG and the degree of substitution. Highly substituted QDs yield half-lives in the 3 h to 8 h range for mPEG-5000-coated QDs^[^^106^^]^.

Although *in vivo* targeting and imaging developed quickly, these techniques are still challenging because of the relatively large overall size (typically >20 nm in hydrodynamic diameter) and short circulation half-lives of QD conjugates.

### Tumor microenvironment for invasion and progression

Cancer progression is not an entirely cell-autonomous process. The invasion is regulated by intrinsic genetic changes in cancer cells as “initiators” of carcinogenesis and by stromal cells as “promoters”. Human cancer is especially complex because it evolves over a long time course and shows a multitude of molecular, cellular, and architectural heterogeneity. Neither the studies at purely molecular and cellular levels, nor the studies at the purely clinical level can decipher the co-evolution of a cancer microenvironment. Such co-evolution of cancer microenvironment has long been underappreciated because of the lack of appropriate technology platforms to reveal the dynamic spatiotemporal processes. QDs, can be good delivery nanocarriers to tumors *in vivo* for multi-parameter imaging given their relatively large surface areas which can be conjugated with more than one targeting ligand such as novel tumor-specific antibody fragments, growth factors, peptides, and small molecules, with the ultimate goal of guiding therapy selection and predicting response to therapy. This nanoplatform approach will enable the simultaneous detection and measurement of several biomarkers, which may lead to better signal/contrast than QDs modified with only one type of targeting ligand. These properties are also very suitable for investigating the co-evolution of cancer cells and tumor microenvironment at the architectural level, a key issue in studying the mechanisms of cancer progression and in developing more specific targeting therapeutic approaches.

Since QDs were first used to detect F-actin in mouse fibroblasts^[^^12^^]^, numerous studies have been conducted for cancer invasiveness, lymphocytes homing, and embryogenesis, confirming that QDs are potential fluorescent probes for *in vivo* imaging of lymph nodes and tumors^[^^94^^]^. A new approach based on multiphoton microscopy techniques and QD nanotechniques was established by Stroh et al.^[^^51^^]^ to differentiate the tumor vessels from the perivascular cells and the matrix. With this approach, the tumor vessels can be identified from solid tumors clearly and vividly.

**Figure 3:**
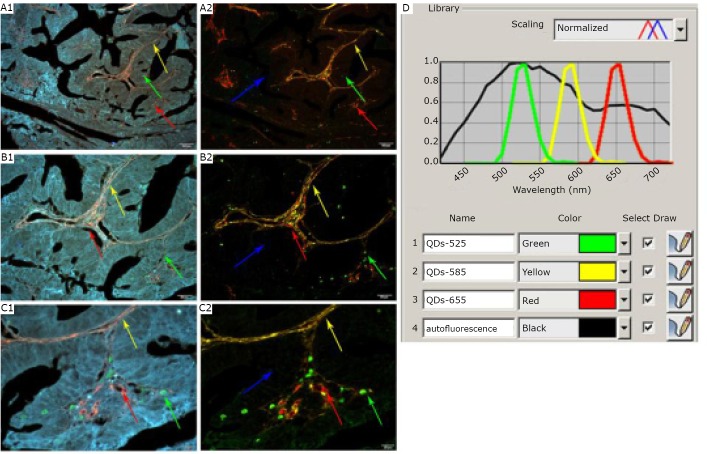
The establishment of multiplexed QD imaging and spectrum analysis. A1, B1, and C1: Infiltrating macrophages (Green arrows), type IV collagen (Yellow arrows), and neovessels (Red arrows) are labeled simultaneously in gastric cancer tissues with nanoprobes QDs-525, QDs-585, and QDs-655, respectively. A2, B2, and C2: Corresponding unmixed image of A1, B1, and C1 obtained by spectrum analysis with differentiable autofluorescence (Blue arrows). D: QD emission spectra and tissue autofluorescence data used for unmixed image. Magnification: ×100 (A1 and A2), ×200 (B1 and B2), and ×400 (C1 and C2); Scale bar: 100 mm (A1 and A2), 50 mm (B1 and B2), and 20 mm (C1 and C2). Reproduced with permission from [103].

**Figure 4:**
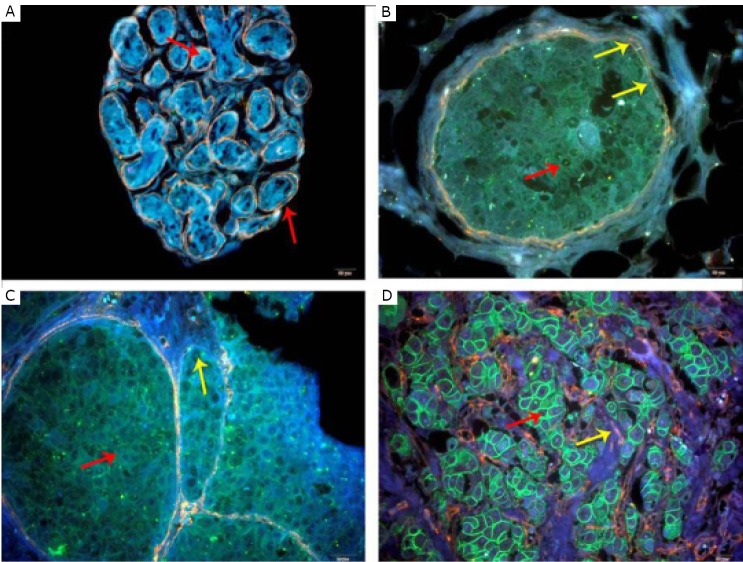
Double-color imaging was used in BC over different levels of HER2. A: Benign breast tumor, no HER2 expression, and intact ECM (Red arrow). B: BC with HER2 (+) (Red arrow), ECM becomes unsmooth and thin (Yellow arrows). C: HER2 (2+) and moderate green fluorescence (Red arrow). ECM becomes significantly degraded (Yellow arrow). D: HER2 (3+), strong green fluorescence (Red arrow) and complete ECM degradation (Yellow arrow). (Magnification: ×, scale bar =20 µm). Reproduced with permission from [69].

Our group recently confirmed the benefit of QD-based multiplexed imaging and spectrum analysis technology to study the co-evolution of cancer cells and tumor stroma by type IV collagen, tumor angiogenesis, macrophage infiltration, and tissue destructive proteolytic enzyme MMP9^[^^107^^,^^108^^]^, which revealed the related molecular features of tumor microenvironment during cancer invasion^[^^108^^]^ (**Figure 3**). Four invasive patterns with distinctive cancer cell-stroma interactions were identified, namely, washing, amoeba-like, polar, and linear patterns. Another research on QDbased doublecolor imaging of HER2 on BC cells and the type IV collagen in the ECM also showcase the dynamic processes of BC invasion (**Figure 4**)^[^^69^^]^.

In our previous *in vivo* imaging of liver cancer xenograft models^[^^102^^]^, the heterogeneous AFP distribution was reflected by QD probes, which showed that the QD fluorescence per field was lower at the center than in the periphery of the tumor, indicating the heterogeneous proliferation in different tumor areas. This phenomenon may provide essential information on “safe” tumor margins to guide surgical resection in clinical practice. If the surgical resection is not wide enough to eradicate the tumor at the margin with strongest proliferation and invasion potentials, the clinical outcome will remain poor, although 99.99% of the cancer cells have been eliminated^[^^109^^]^.

Doxorubicin (Dox)-conjugated QDs are used to target alveolar macrophages and inflammation. A study showed the absence of significant effects of inflammatory injury parameters (albumin leakage, proinflammatory cytokines, and neutrophil infiltration) after QD-Dox treatment compared with Dox *in vivo*, demonstrating that nanoparticle platforms provide targeted macrophage-selective therapy for the treatment of pulmonary disease^[^^110^^]^. Another group found that QDs impair macrophagic morphology and the ability of phagocytosis by inhibiting Rho-associated kinase signaling, which will contribute to a better understanding of the tumor microenvironment^[^^111^^]^. Hence, QD-based nanotechnology can reveal and detect the role and function of the tumor microenvironment and can be used for novel targeting therapy.

## Application of QD-based Multimodality Biomedical Molecular imaging

In general, molecular imaging modalities include molecular MRI, magnetic resonance spectroscopy (MRS), optical bioluminescence imaging, optical fluorescence imaging, targeted ultrasound, single-photon-emission computed tomography (SPECT), and positron emission tomography (PET). Among all the molecular imaging modalities currently available, no single modality is perfect and sufficient to obtain all the necessary information^[^^112^^]^. The accurate quantification of the fluorescence signals in living subjects, particularly in deep tissues, is quite difficult. MRI has high resolution, but with low sensitivity. Radionuclide-based imaging techniques have very high sensitivity, but with relatively poor resolution. The combination of multiple molecular imaging modalities can offer synergistic advantages over any modality alone. Thus, given that biomedical imaging technologies are now well-developed, cancer imaging should receive a new dimension and momentum with the design and synthesis of suitable multimodal probes based on QDs. This condition appears achievable in the context of the rapid growth in the field of QDs and the wealthy information on the molecular mechanisms of cancer.

At present, various hybrid systems that combine two or more of these imaging modalities are under active investigation^[^^113^^]^. With a size approximately 100 to 10,000 times smaller than human cells, QDs can offer unprecedented interactions with biomolecules on the surface of and inside cells, which may revolutionize disease diagnosis and treatment.

Nanotechnology has touched every single modality of the molecular imaging arena. Despite the great potential of optical imaging, accurately quantifying any QD signal in deep tissues based on fluorescence alone is challenging. This intrinsic limitation is now being addressed by developing QD-based probes that combine multiple molecular imaging modalities onto a single QD nanoparticle platform. Indeed, the tens to hundreds of square nanometers of surface area of QDs represent invaluable assets for surface integration of paramagnetic or radioactive agents that enable three-dimensional tomography techniques. QD-based nanoplatforms are mainly integrated with MRI and PET. For example, Mulder et al.^[^^114^^,^^115^^]^ successfully targeted tumor angiogenesis by fluorescence and MRI imaging based on the MR-fluorescence bimodal QDs. This approach was extended to QD-based bimodal probes contained in a silica nanoparticle to improve biocompatibility^[^^116^^]^. It was also successfully applied in lymphatic imaging^[^^117^^]^ or combined with other imaging modalities^[^^118^^]^. In another group, QD-based probes enabled PET after chelation with ^64^Cu^[^^100^^]^. Such dual-modality probes have provided unique quantitative information pertaining to tumor-targeting efficiency and *in vivo* kinetic biodistribution^[^^119^^]^, thus helping optimize the next generation of QD probes for *in vivo* imaging. This design of QD-based imaging agents will allow simultaneous and quantitative PET detection of multiple spectrally distinct targets. Nuclear spin labels for MRI^[^^120^^]^ or SPECT/CT contrast radionuclide could also be incorporated into the QDs. A further step could involve transmission electron microscopy imaging of the precise localization of QDs within cells and tissues ^[^^121^^]^. Hence, QDs have the potential to provide information over a wide range of length scales (**Table 2**).

**Table 2:** Applications of QD-based multimodality imaging for tumor.

Probes	Modality	Size (nm)	Application	References
^64^Cu-DOTA to CdTe/ZnS (QD705)	PET/optical	ND	Tumor vasculature imaging	[100,122,123]
^64^Cu-DOTA to CdSe/ZnS (QD525, QD800)	PET/optical	ND	Quantitative biodistribution in living mice	[124]
Gd-DOTA to CdSeTe/CdS/glutathione	MRI/optical	7 to 10	Lymph node imaging of mouse	[125]
Iron oxide and CdSe/ZnS micelle	MRI/optical	6.7	Simultaneous targeted drug delivery and dual-mode imaging of tumor tissues by near-infrared fluorescence and NMR spectroscopy	[126]
Resolve Al-Gd and CdSe/ZnS micelle	MRI/optical	18	Tracing blood circulation *in vivo*	[127]
Gd-lipid in coating and CdSe/ZnS/silica	MRI/optical	15	Tumor angiogenesis imaging	[115]
MnCdTeSe/CdS	MRI/optical	4 to 50	Pancreatic cancer imaging	[128]

Meanwhile, due to the size and structural similarities between imaging and therapeutic nanoparticles and the multifunctionality and enormous flexibility of QDs, they can allow the integration of therapeutic components, targeting ligands, and multimodality imaging labels into one entity, termed “nanomedicine,” for the ideal target; QDs also show great potential for treating tumors in animal models^[^^129^^]^. Bagalkot et al.^[^^130^^]^ reported on a ternary system composed of a QD, an aptamer, and the small molecular anticancer drug Dox with three functions integrating targeted imaging and therapy with sensing of drug release. In this system, fluorescence of QDs was quenched by Dox, and Dox was quenched by the double-stranded RNA aptamers through fluorescence resonance energy transfer. As a result, the gradual release of Dox enhanced the local anticancer effects, and the fluorescence of QDs provided a method to sense the release of the drug and a versatile nanoscale scaffold to develop multifunctional nanoparticles for siRNA delivery and imaging. Nowadays, RNA interference has broad applications, ranging from functional gene analysis to targeted therapy of cancer^[^^131^^-^^136^^]^. Yezhelyev et al.^[^^137^^]^ constructed a multifunctional nanoparticle for siRNA delivery based on QDs, which showed highly effective and safe RNA interference, as well as fluorescence contrast. It can improve gene silencing activity by 10 to 20 folds, and can reduce cytotoxicity by 5 to 6 folds, compared with current siRNA delivery agents. In addition, QDs are inherently dual-modality optical and electron microscopy probes, allowing real-time tracking and ultrastructural localization during transfection.

Fluorescence imaging and photodynamic therapy (PDT) are used in advanced clinical trials for the efficient detection and cancer treatment. Compared with chemotherapy and radiation therapy, these methods offer selective therapy with the immune system and normal cells remaining intact. QDs are the most promising property for PDT with exceptional photostability^[^^138^^,^^139^^]^_._

## Challenges and Future Prospects

### Nanotoxicology

Although QDs have great potential for biomedical imaging and detection, toxicological and pharmacological issues mainly from heavy metal and colloidal instability limit the advancement toward the diagnosis and therapy of cancer and other diseases^[^^140^^,^^141^^]^. These concerns may not hinder the development of the applications *in vitro*; however, they serve as great barriers for human application for *in vivo* cancer imaging. Efforts have been exerted to generate novel QDs based on their components, sizes, surface coatings, and valences to minimize toxicity and maximize detection efficiency^[^^36^^,^^142^^-^^148^^]^. However, problems such as coating shell degradation caused by the modification of QDs should be considered^[^^57^^]^. Alternatively, nonspecific accumulation by the RES, including the liver, spleen, and lymphatic system, should also be observed^[^^30^^,^^57^^,^^96^^,^^140^^]^. In addition, immune response^[^^149^^]^ and genotoxic effect^[^^150^^]^ have been reported. Several studies have shown that QDs less than 5 nm in size can be removed by the kidney^[^^30^^,^^141^^]^. Thus, considering the biosafety for *in vivo* applications, long-term toxicological and pharmacokinetic investigations involving degradation, excretion, persistence, and immune response of QDs should be systematically assessed.

Considering the toxicity of Cd, Se, Zn, Te, Hg, and Pb, several low-toxicity QDs have been developed as substitutes^[^^91^^,^^151^^,^^152^^]^. For example, low toxicity is achieved by replacing Cd with Zn. Such QDs are also less sensitive to environmental changes, such as thermal, chemical, and photochemical disturbances. These doped QDs have color-tunability with good quantum efficiency and are promising candidates for future efforts to lower QD-based cytotoxicity. They also have narrow emission spectra (45 nm to 65 nm full width at half maximum) and can cover most of the visible spectral window. In the near future, doped QDs that emit in the NIR region will be developed. Extensive scrutiny and research on the toxicity profiles will be needed before QDs can be employed in any medical procedure. Further studies are also needed to investigate the clearance mechanism of QDs from living systems.

### Design and Generation of Biocompatible and Biodegradable Nanoparticles

QD-based *in vivo* imaging and targeting studies are limited due to nonspecific organ uptake and RES scavenging, namely, the relatively large size (15 nm to 30 nm) and short circulation half-life in the blood vascular system. Thus, various current groups attempt to prolong the circulation time of QDs by attaching passivating molecules, such as PEG, and by controlling the overall charge of the particles to prevent their adsorption to the plasma proteins^[^^69^^,^^116^^,^^126^^]^. Alternatively, clearance from the body is a prerequisite to the clinical use of any contrast agent. An intriguing recent finding suggests a size threshold of 5 nm to 6 nm in diameter, below which the QDs cannot escape the liver and be cleared through the kidneys^[^^153^^]^.

### Reproducibility, Reliability, and Comparability of QDs

The present clinical applications of QDs are strongly limited by the deficient amount of data on their reproducibility and comparability, as well as on their potential for quantification. Different functionalized QDs from various sources will have different fluorescence quantum yields based on variable materials and surface chemistries. Thus, the derivation and establishment of quality criteria for these materials of different functionalized QDs is the essential initial step^[^^154^^]^.

## Conclusions

QDs are technological marvels with characteristics that may revolutionize cancer diagnosis and treatment. Currently, QDs are widely used *in vitro*, such as in detecting cancer biomarkers in molecular pathology, revealing cancer invasion, focusing on the tumor environment, and providing a novel approach for improving tumor heterogeneity understanding, diagnosis, classification, and treatment of cancer. However, complex in vivo studies still result in the difficult identification of the dominant and compensation mechanisms of tumor invasion and microenvironment^[^^4^^]^.

In clinical settings, optical imaging is relevant for tissues close to the surface of the skin, accessible by endoscopy, and during intraoperative visualization. The future of nanomedicine lies in the multifunctional nanoplatforms, which combine therapeutic components and multimodality imaging. The ultimate goal is for nanoplatform-based agents to allow the efficient and specific in vivo targeted delivery of drugs without systemic toxicity, and the dose delivered, as well as the therapeutic efficacy, can be accurately measured noninvasively over time. However, inefficient delivery, potential toxicity, and lack of quantification are also major roadblocks for clinical translation of QDs.
